# Epidemiology of firearm injuries in Sweden

**DOI:** 10.1007/s00068-021-01735-8

**Published:** 2021-07-02

**Authors:** Karolina Nyberger, Eva-Corina Caragounis, Pauline Djerf, Carl-Magnus Wahlgren

**Affiliations:** 1 Department of Molecular Medicine and Surgery, Karolinska Institute, Solna, Sweden; 2 Department of Vascular Surgery, Karolinska University Hospital, SE-171 76 Stockholm, Sweden; 3 Department of Surgery, Institute of Clinical Sciences, Sahlgrenska University Hospital, Sahlgrenska Academy, University of Gothenburg, Gothenburg, Sweden; 4 Department of Surgery, Lund University, Skåne University Hospital, Lund, Sweden

**Keywords:** Firearm injury, Gunshot wound, Epidemiology

## Abstract

**Background:**

Gun violence is a global health problem. Population-based research on firearm-related injuries has been relatively limited considering the burden of disease. The aim of this study was to analyze nationwide epidemiological trends of firearm injuries.

**Methods:**

This is a retrospective nationwide epidemiological study including all patients with firearm injuries from the Swedish Trauma Registry (SweTrau) during the period 2011 and 2019. Registry data were merged with data from the Swedish National Council for Crime Prevention and the Swedish Police Authority.

**Results:**

There were 1010 patients admitted with firearm injuries, 96.6% men and 3.4% women, median age 26.0 years [IQR 22.0–36.3]. The overall number of firearm injuries increased on a yearly basis (*P* < 0.001). The most common anatomical injury location was lower extremity (29.7%) followed by upper extremity (13.8%), abdomen (13.8%), and chest (12.5%). The head was the most severely injured body region with a median abbreviated injury scale (AIS) of 5 [IQR 3.2–5]. Vascular injuries were mainly located to the lower extremity (42%; 74/175). Majority of patients (51.3%) had more than one anatomic injury location. The median hospital length of stay was 3 days [IQR 2–8]. 154 patients (15.2%) died within 24 h of admission. The 30-day and 90-day mortality was 16.7% (169/1010) and 17.5% (177/1010), respectively. There was an association between 24-h mortality and emergency department systolic blood pressure < 90 mmHg [OR 30.3, 95% CI 16.1–56.9] as well as the following injuries with AIS ≥ 3; head [OR 11.8, 95% CI 7.5–18.5], chest [OR 2.3, 95% CI 1.3–4.1], and upper extremity [OR 3.6, CI 1.3–10.1].

**Conclusions:**

This nationwide study shows an annual increase of firearm-related injuries and fatalities. Firearm injuries affect people of all ages but more frequently young males in major cities. One in six patients succumbed from their injuries within 30 days with most deaths occurring within 24 h of hospital admission. Given the impact of firearm-related injuries on society additional research on a national level is critical.

## Introduction

Violence with firearms is considered to be a complex public health issue [1]. About a quarter of a million people worldwide die from firearm injuries annually, the majority associated with homicides [2]. Gun-related deaths affect men and younger people to a greater extent. Rates of firearm deaths vary among countries and across demographic subgroups. The rate of gun-related mortality is higher in the United States than other high-income countries in the world [3]. However, penetrating trauma has become a highly relevant issue also in Europe in recent years [4, 5]. Firearm injuries do not only affect the livelihood of those injured but also cause major impact in terms of financial burden on healthcare systems and society [6].

Research on violence with firearms has been relatively limited considering the burden of disease and there is a need for more studies including data on epidemiology and clinical outcome to further improve management of these injuries [7]. The contemporary experience from a single Scandinavian trauma centre has shown an increase in firearm injuries in recent years [8]. It is important to study how firearm injuries affect health care systems on a national level to develop robust trauma systems improving field triage and identifying patients with a need for trauma centre care. In addition, further research may identify areas to implement injury prevention strategies. The aim of this study was to analyze nationwide trends in firearm injuries including demographics, operative procedures, and patient outcomes.

## Methods

### Study population

This was a retrospective nationwide epidemiological study including all patients with firearm injuries from the national Swedish Trauma Registry (SweTrau) from January 1, 2011 to December 31, 2019. There were 71,879 trauma patients registered during the study period, of which 1010 patients were identified with firearm injuries (1.4%). The study was approved by the Ethical Review Agency (2019-05863).

### Study aims

The primary aim was to investigate nationwide epidemiology of firearm injuries during the study period. Secondary aims were to assess anatomical distribution of injuries, operative procedures, and patient outcome.

### Data sources

Data on demographics as well as anatomical localization of injury, surgery, and mortality were extracted from the national trauma registry, SweTrau, which was founded in 2011 [9]. SweTrau is the only nationwide trauma database, covering 84% of the trauma receiving hospitals in Sweden. The SweTrau database follows “*The Utstein Trauma Template for Uniform Reporting of Data Following Major Trauma; Data Dictionary*”, which represent a uniform set of variables considered most important for comparing trauma systems and outcomes in Europe. Data access for this study was approved by the registry steering committee. Open access data regarding the total number of deaths due to lethal violence were collected from the Swedish National Council for Crime Prevention and data on the annual number of confirmed shootings from the Swedish Police Authority (reports started November 2016).

### Inclusion and exclusion criteria

Patients of all ages and genders, admitted with firearm injuries, were included in the study (*n* = 1010). Patients admitted to the reporting hospital more than 24 h after injury and patients declared dead before hospital arrival was excluded. To be registered in SweTrau, patients have to fulfill at least one of the following inclusion criteria: Trigger a trauma team activation, have a New Injury Severity Score (NISS) > 15, or have a NISS > 15 and be transferred from another hospital within 7 days. Trauma team activations are either Level 1 triggers whereby a large team resucitates trauma patients with physiological impairment and/or with obvious injury, or Level 2 triggers whereby a limited team assesses patients without physiological impairment subjected to a specific mechanism of injury. Injuries that were not caused by firearms were excluded from further analysis (*n* = 70,869). Missing values for specific variables were imputed for some variables and are reported below.

### Definitions

Firearm injuries were defined using the International Classification of Diseases (ICD) codes for injury mechanisms: X93-Assault by handgun X94-Assault by rifle, shotgun, and larger firearm; and X95-Assault by other and unspecified firearm.

According to the Swedish National Council for Crime Prevention, a confirmed shooting is defined as an occasion when projectiles with a gunpowder-charged weapon have been fired, and there are traces of bullets, cartridge cases, or damage to materials or people related to the shooting, or there is more than one independent eyewitness to the firing. The shooting must also be illegal and intentional. Furthermore, the mortality data from the Swedish National Council for Crime Prevention on deaths by firearm injuries were defined by all deaths caused by illegal shootings. Hence, the data included both individuals confirmed dead at the scene and individuals confirmed dead after hospital arrival.

The anatomical classification according to the Abbreviated Injury Scale (AIS) was used to classify anatomical injury location [10]. To minimize missing Revised Trauma Score (RTS) values, unpalpable radial pulse was converted to a systolic blood pressure below 90 mmHg [11].

### Statistical analysis

Data were presented as median with interquartile range (IQR). Descriptive statistics were performed for patient characteristics and outcomes. Univariate analyses of binary and nominal variables were performed using cross-tabulations and values were reported for the Pearson’s chi-square and Fisher’s exact tests. Univariate analysis and multivariable logistic regression analyses were performed to identify risk factors for 24-h and 30-day mortality post-trauma admission. In the logistic regression model, age, gender, NISS, emergency department systolic blood pressure, and AIS score of injured body regions were all included as covariates. All covariates mentioned were included in both the univariate and multivariate analyses except for NISS in the multivariate analysis due to multicollinearity. The Poisson regression model was used to analyze trauma trends over the years. Kaplan–Meier method was used for presentation of survival. *P* value < 0.05 was considered significant. Analysis was performed with IBM^®^ SPSS Statistics V22.0.

## Results

### Patient demographics

**Fig. 1:**
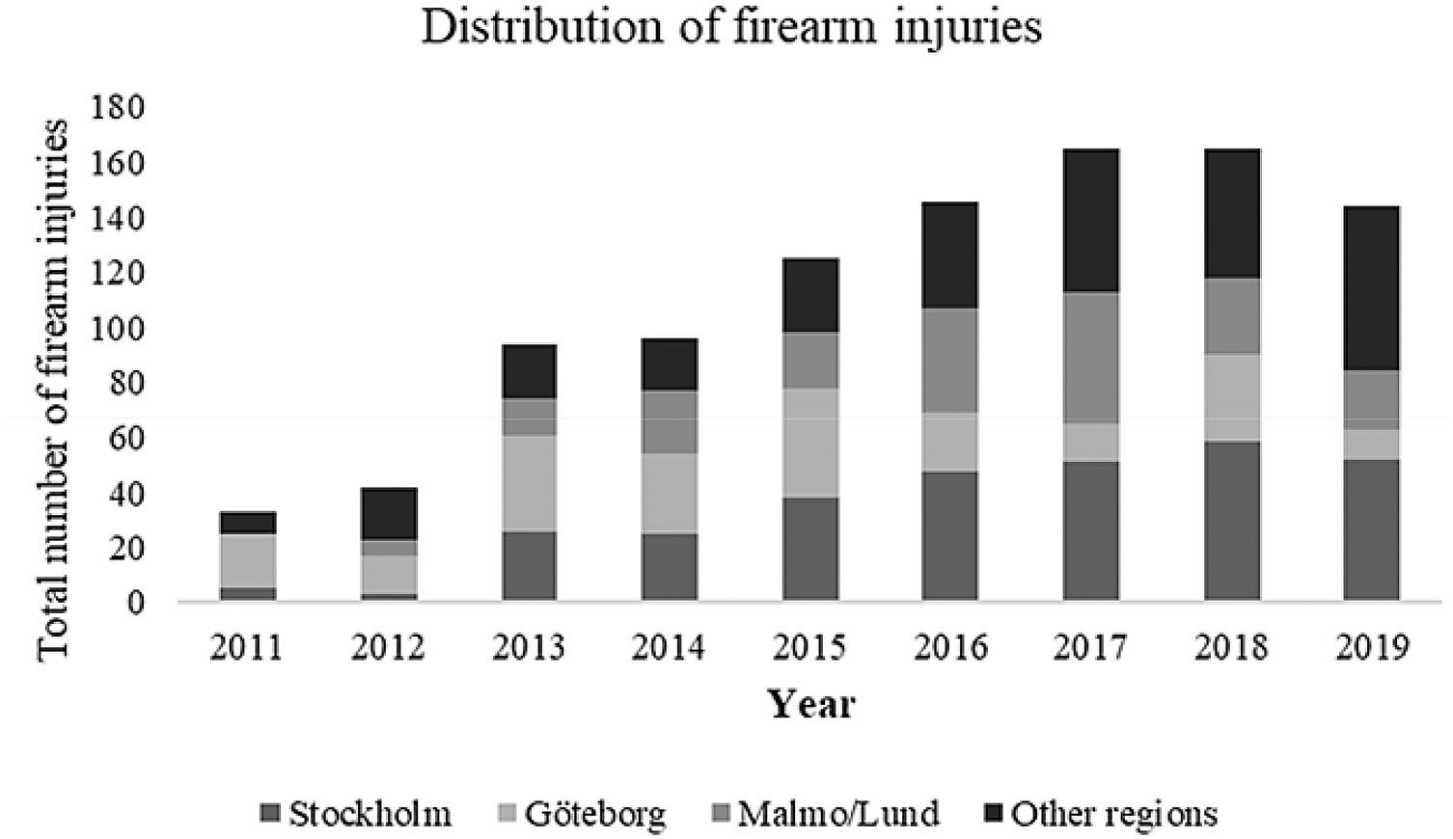
Annual number of firearm injuries nationwide with the distribution in the three most populated regions in relation to other regions

This study included 1010 patients admitted with firearm injuries, 96.6% men (*n* = 976) and 3.4% women (*n* = 34) with a median age of 26.0 years [IQR 22.0–36.3] (Table 1). The median ISS was 9 [IQR 1–18] and median NISS was 11 [IQR 3–26]. The most common injury intent was assault (76.9%; *n* = 777). Firearm injuries dominated in the younger population where the age group 21–30 years (*n* = 442) was most common, followed by age group < 20 years (*n* = 194) and 31–40 years (*n* = 151). There were fewer injuries in the population > 70 years (*n* = 32). Nevertheless, there was an increase in all age groups over time (*P* < 0.001). The number of firearm injuries has increased over the years (*P* < 0.001) (Fig. 1). This increase was also seen for women during the study period (*P* = 0.001). The confirmed number of annual shootings on a national level in relation to the number of trauma admissions from 2017 to 2019 is displayed in Table 2.

**Table 1:** Patient demographics and clinical data with prehospital and emergency department parameters

Demographics and clinical data		*n*
Age (years)*	26 [22.0–36.3]	1000
Men	976 (96.6%)	1010
Women	34 (3.4%)	1010
Injury intent		
Accident	45 (4.5%)	1010
Self-harm	88 (8.7%)	1010
Assault	777 (76.9%)	1010
Other	30 (3.0%)	1010
Unknown	70 (6.9%)	1010
ISS	9 [1–18]	991
NISS	11 [3–26]	991
NISS ≤ 15	4 [2–9]	603
NISS 16–24	18 [17–22]	114
NISS ≥ 25	34 [27–57]	274
Prehospital SBP (mmHg)	130 [110–144]	562
Prehospital GCS	15 [15–15]	660
Prehospital RR/min	20 [18–24]	521
ED SBP (mmHg)	135 [119–150]	821
ED GCS	15 [15–15]	842
ED RR/min	19 [16–22]	574

*ISS* Injury Severity Score, *NISS* New Injury Severity Score, *ED* emergency department, *SBP* systolic blood pressure, *GCS* Glasgow–Coma Scale, *RR* respiratory rate

*Median [IQR]

**Table 2:** Confirmed illegal shootings on a national level in relation to hospital trauma admissions and mortality, including patient fatalities not reaching hospitals

Year	Confirmed illegal shootings*	Trauma admissions	Total mortality illegal shootings**	In-hospital mortality***
2011	NA	33	17	3
2012	NA	42	17	5
2013	NA	94	25	13
2014	NA	96	28	16
2015	NA	125	33	20
2016	NA	147	30	21
2017	324	164	40	25
2018	306	165	43	31
2019	334	144	45	29

*NA* data not available

*Data from the Swedish Police Authority; registration from 2017

**Data from the Swedish National Council for Crime Prevention

***Data from the Swedish National Quality Registry for Trauma (SweTrau)

The majority of firearm injuries (71.2%) occurred in the three most populated regions (Stockholm 30.4%; Gothenburg 21.3%; Malmo/Lund 19.5%) in the country. A comparison of the annual distribution of firearm injuries between Sweden’s three largest regions and other regions is included in Fig. 1.

### Clinical data

Prehospital and emergency department (ED) vital signs are displayed in Table 1. Prehospital and ED systolic blood pressure (SBP) < 90 mmHg or no palpable radial pulse was seen in 20.3% (142/701) and 15.3% (143/934), respectively. There were 13.0% (98/752) prehospital cardiac arrests. Prehospital and ED intubation was performed in 12.4% (92/742) and 21.6% (197/910), respectively.

The most common prehospital transportation was ground ambulance, 68.7% (694/1010); 5.3% (*n* = 54) arrived with helicopter, 6.8% (*n* = 69) with private transportation, 4.3% (*n* = 43) walking, 2.5% (*n* = 25) with police, 0.3% (*n* = 3) with other transportations, and 12.1% (*n* = 122) used unknown transportation.

### Patient injury data

The annual distribution of injuries in each anatomical zone is presented in Table 3. The most common anatomical injury location was lower extremity (29.7%, *n* = 585) followed by upper extremity (13.8%, *n* = 273), abdomen (13.8%, *n* = 272), chest (12.5%, *n* = 246), head (11.1%, *n* = 219), face (5.6%, *n* = 111), spine (5.6%, *n* = 111), neck (2.9%, *n* = 58), and unknown region (5.0%, *n* = 98). The head was the most severely injured body region with a median AIS of 5 [IQR 3.25–5]. The majority of patients (51.3%, *n* = 518) had more than one anatomic injury location where the combination of lower extremity and abdomen dominated (15.2%, *n* = 154). In patients arriving in the ED with SBP < 90 mmHg or no palpable radial pulse (*n* = 143), anatomic injury location to the chest dominated (69.2%, 99/143), followed by the lower extremity (44.8%, *n* = 64) and the abdomen (42.7%, *n* = 61).

**Table 3:** The annual distribution of injuries in anatomical zones

	Head	Face	Neck	Thorax	Abdomen	Spine	Upper extremity	Lower extremity	Unspecified
2011	5	3	3	9	13	7	8	17	4
2012	11	3	1	8	9	1	9	21	5
2013	25	11	1	15	21	9	23	60	12
2014	17	7	12	25	28	7	26	49	9
2015	32	17	7	31	38	14	41	67	10
2016	29	18	7	37	38	14	42	91	10
2017	41	17	13	43	53	21	41	96	13
2018	36	20	9	39	37	25	46	98	14
2019	23	15	5	39	35	13	37	86	21
Total (*n*)	219	111	58	246	272	111	273	585	98

There was a total of 175 registered vascular injuries. Most vascular injuries were located to lower extremity (42%, *n* = 74) followed by abdomen (18%, *n* = 32), chest (18%, *n* = 32), and upper extremity (10%, *n* = 18).

### Surgical procedures related to firearm injuries

There were in total 1249 primary surgical procedures that were performed in 881 patients (Table 4). Wound-related surgical procedures dominated, followed by fracture surgery. There were in total 146 laparotomies and 80 thoracotomies. For patients arriving in the ED with SBP < 90 mmHg or no palpable pulse, 37.1% (53/143) underwent thoracotomy and 19.6% (*n* = 28) laparotomy; 14.7% (*n* = 21) experienced both laparotomy and thoracotomy. Thoracotomy was performed in 38.8% (38/98) of patents with prehospital cardiac arrest and laparotomy in 17.3% (*n* = 17).

**Table 4:** Types of surgical procedures performed due to firearm injuries

Type of surgical procedure	Number of patients (*n*)
Wound suture/revision	413
Fracture surgery	177
Removal foreign body	178
Laparotomy	146
Chest tube	118
Thoracotomy	80
Open vascular	55
Fasciotomy	45
Endovascular	15
Craniotomy	17
Neck exploration	5
No surgical procedure	129

### Patient outcome

**Fig. 2:**
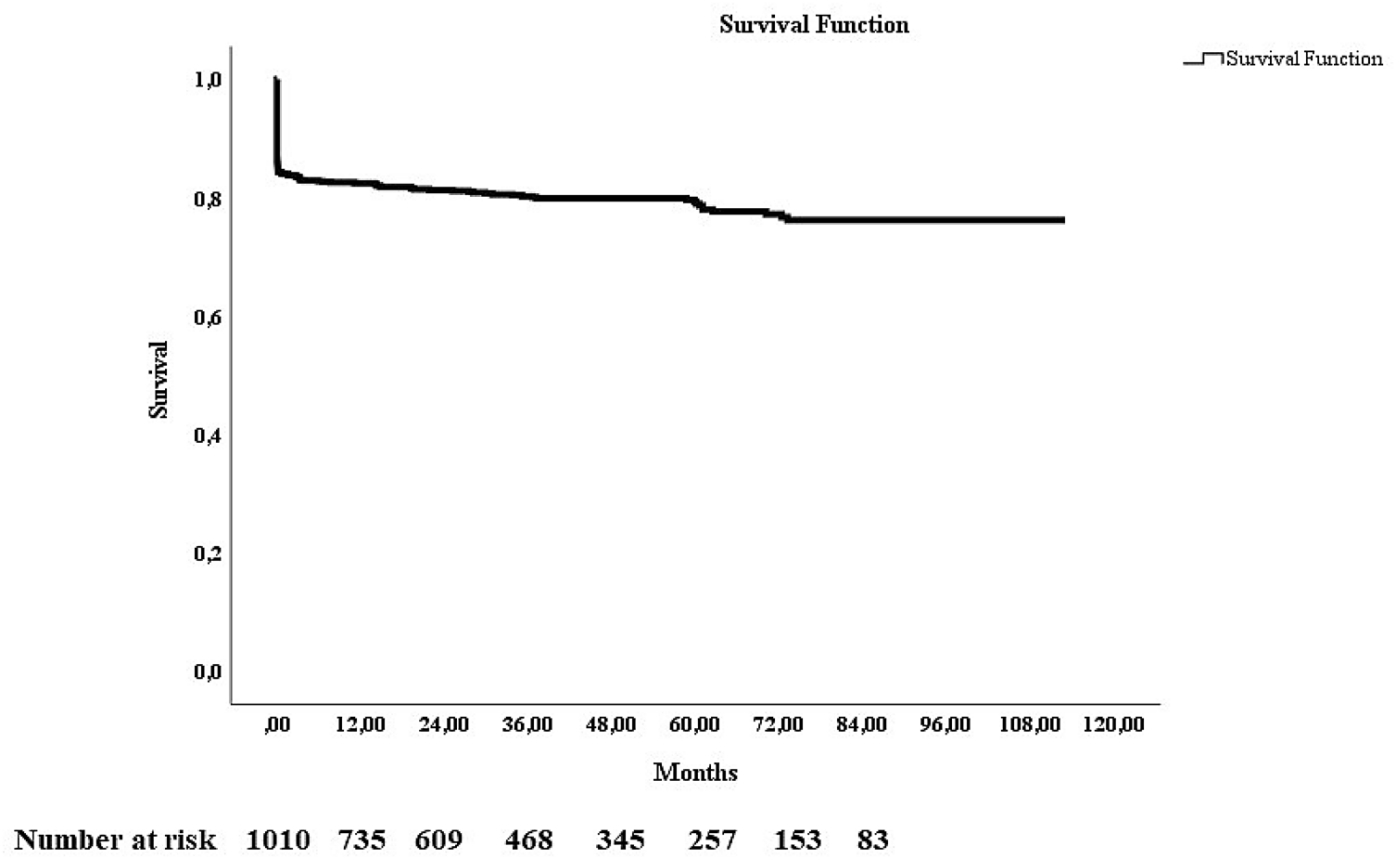
Kaplan–Meier long-term survival curve after hospital admission from firearm injury

The median number of days on mechanical ventilation was 2 ([IQR 1–4], *n* = 229). The median hospital length of stay was 3 days ([IQR 2–8], *n* = 961). One hundred fifty-four patients (15.2%) died within 24 h of admission of whom 90 had prehospital cardiac arrest. In patients arriving with cardiac arrest only 8.2% survived the first 24 h. The 30-day and 90-day mortality was 16.7% (169/1010) and 17.5% (*n* = 177), respectively. The total number of deaths was 213 (21.1%) upon the day of data extraction and the median follow-up time was 33.3 months [IQR 10.5–60.9]. The long-term survival curve is displayed in Fig. 2.

The total firearm-related fatalities in the country from The Swedish National Council for Crime Prevention, also including patient fatalities not reaching hospitals, in relation to in-hospital mortality per year are shown in Table 2. The majority of patients were discharged from hospital directly to their homes (60.4%, 610/1010); 163 patients (16.1%) to the morgue, and 65 patients (6.4%) were discharged to rehabilitation.

Univariate analysis and multivariable logistic regression analyses were performed to identify risk factors associated with 24-h and 30-day mortality (Table 5). In the univariate analysis, ED SBP < 90 mmHg, NISS ≥ 25, as well as the injuries to the head, neck, chest, abdomen, and upper extremity with AIS ≥ 3 were associated with both 24-h and 30-day mortality. In the multivariable analysis, there was an association between 24-h mortality and ED SBP < 90 mmHg [OR 30.3, 95% CI 16.1–56.9] as well as the following injuries with AIS ≥ 3; head [OR 11.8, 95% CI 7.5–18.5], chest [OR 2.3, 95% CI 1.3–4.1], and upper extremity [OR 3.6, CI 1.3–10.1]. A similar injury pattern with ED SBP < 90 mmHg [OR 37.7, 95% CI 19.4–73.1] as well as injuries with AIS ≥ 3 to the head [OR 12.3, 95% CI 8.0–19.1], face [OR 5.0, 95% CI 1.5–17.0], chest [OR 2.9, 95% CI 1.7–5.0], and upper extremity [OR 3.3, CI 1.1–10.0] were associated with 30-day mortality.

**Table 5:** Risk factors associated with mortality at 24-h and 30-day post-trauma admission

	Odds ratio [95% confidence interval]				
	24 h		30-day	24 h	30-day
	Univariate logistic regression			Multivariable logistic regression	
Age	1.01 [1.00–1.02]		1.02 [1.01–1.03]	1.02 [1.00–1.04]	1.03 [1.01–1.05]
Female gender	1.20 [0.49–2.94]		1.28 [0.55–3.00]	1.16 [0.48–2.80]	1.01 [0.42–2.44]
ED SBP < 90 mmHg	28.27 [15.49–51.58]		31.60 [17.30–57.70]	30.29 [16.12–56.92]	37.67 [19.42–73.09]
NISS					
16–24		2.01 [0.82–4.90]	2.11 [0.91–4.90]		
≥ 25		21.57 [12.87–36.16]	23.48 [14.29–38.60]		
Body region with AIS ≥ 3					
Head		7.17 [5.11–10.06]	9.20 [6.42–13.17]	11.79 [7.52–18.47]	12.34 [7.98–19.08]
Face		0.81 [0.18–3.58]	3.51 [1.23–10.02]	2.06 [0.44–9.61]	5.00 [1.47–16.98]
Neck		7.71 [3.34–17.79]	10.10 [4.16–24.47]	5.17 [0.60–44.19]	4.83 [0.51–45.64]
Chest		4.35 [3.30–5.72]	4.63 [3.50–6.13]	2.33 [1.33–4.09]	2.92 [1.70–5.02]
Abdomen		2.12 [1.51–2.97]	2.22 [1.59–3.09]	1.54 [0.89–2.68]	1.66 [0.98–2.82]
Spine		1.99 [0.94–4.22]	2.03 [0.98–4.22]	1.49 [0.47–4.68]	1.82 [0.60–5.52]
Upper extremity		2.38 [1.26–4.51]	2.10 [1.11–3.98]	3.61 [1.28–10.16]	3.34 [1.11–10.00]
Lower extremity		0.58 [0.40–0.86]	0.57 [0.39–0.82]	0.53 [0.29–0.99]	0.52 [0.28–0.95]

## Discussion

Violence due to firearms is a major global public health issue and frequently contributes to premature deaths with homicides being the most common cause [1–3]. This nationwide study confirms an annual increase of firearm-related injuries in Sweden that may have reached a plateau in the past 2 years. Moreover, there were approximately twice as many reported illegal shootings in the nation compared to firearm-related trauma admissions. Firearm injuries affect people of all ages, but have a disproportionate impact on young males in major cities. Furthermore, injuries to the lower extremity were most common and the dominating surgical procedures were wound-related and fracture surgery. Firearm-related fatalities have overall increased annually in the nation. One in six patients succumbed from their injuries within 30 days with most deaths occurring within 24 h of admission to hospital.

Recent studies suggest that firearm injuries have an upward trend in high-income countries, resulting in increased hospitalization [12, 13]. Avraham et al*.* showed an increase in firearm-related injuries treated in the US which are primarily instigated by injuries to young adults causing significant overall mortality of 7.8% [14]. Our study was no exception and showed an annual rise in the total number of GSW. In general, with some annual variations, there was an increase in firearm injuries both in populated and less populated regions. It has been suggested that men are more likely to be hospitalized for GSW but less likely to die [15]. Despite a few cases of women being reported, we noted an annual increase of firearm-related injuries in women that needs further analysis. We could not find any associations between gender and outcome which might be explained by the low number of women reported in this registry. Previous studies have shown a higher rate of assault by intimate partners, increased incidence of pre-existing mental illness, more frequent injury to the extremities, and a shorter ICU stay in women [15, 16]. Moreover, it has been suggested that firearm injuries due to gun violence are overrepresented in some socioeconomic and demographic subgroups which were not possible to investigate in-depth in this study [17].

There were about 20% of patients with prehospital hemodynamic instability. These patients had injuries mainly located to the chest, lower extremity, and abdomen. The majority underwent emergent laparotomy or thoracotomy. Hemodynamic instability was not surprisingly associated with mortality. This is in line with a previous study from the National Trauma Data Bank (NTDB), showing that every SBP drop of 10 mmHg increased mortality with 4.8% in patients with SBP of 110 mmHg and lower [18]. In a previous Swedish study, Ghorbani et al*.* showed that hemorrhage was the cause of deaths in 70% of all penetrating trauma [19]. This suggest that death due to hemorrhage still remains the most common preventable cause of death after firearm injuries, and early recognition of shock is essential [20, 21]. Furthermore, additional analysis of firearm-related trauma deaths may identify preventable measures and areas of improvement in clinical management [20].

This study found the extremities to be the dominating anatomic region for firearm injuries. This is supported by other studies, showing that the extremities are the most commonly injured anatomic regions in non-fatal GSW [22]. More than 40% of vascular injuries were located to the lower extremities. Compressible bleeding and the more frequent use of tourniquets may explain hemodynamic stability in this patient group. In a study by Berg et al., gunshot-related fractures occurred in one-fifth of patients which increased the risk of vascular and nerve injury [22]. The majority of injuries in this study were located to more than one body region where the lower extremity combined with the abdomen was most common. In patients with hemodynamic instability and GSWs to the lower quadrants of the abdomen and groins, there may be a need for exploratory laparotomy to achieve rapid proximal vascular control and haemostasis. There was no anatomic body region that increased in numbers over time. The most severely injured body region was the head which was associated with 24-h and 30-day mortality.

A retrospective registry review of patients treated for GSW at an urban major trauma center in the US showed that 75% of patients admitted to hospital needed at least one surgical procedure during hospitalization [23]. Moreover, it has been suggested that systolic hypotension, hypothermia, and tachycardia are associated with an increased likelihood of surgical intervention [24]. In our population, 87% of patients admitted to hospital underwent one or more surgical procedure. The most common procedure was wound -related surgery (30.0%), followed by removal of foreign body (12.9%) and fracture surgery (12.8%). The number of performed laparotomies and thoracotomies nationwide are lower compared to a previous study from an urban university trauma centre which potentially could be explained by different experiences and management protocols [8].

American College of Surgeons (ACS) National Trauma Data Bank reported a 30-day mortality rate of 15.3% after firearm injuries [25]. The outcome is similar in this study with a 24-h mortality of 15.2%, and 30- and 90-day mortality of 16.7 and 17.5%, respectively. This supports the notion that firearm-related mortality occurs most frequently within 24 h. High NISS and hemodynamic instability are important mortality predictors, and head injury is especially lethal and confirmed by other studies [8, 26, 27].

There are limitations inherent to retrospective national registry studies including limited variables and missing data for specific variables. However, population-based data reflects real-life experience and the results may be applicable in a wider perspective. The SweTrau inclusion criteria may underestimate the number of firearm injuries, but these criteria are according to the established templates for uniform reporting of data following major trauma [28]. Additionally, patients that were already dead at the scene due to gun violence were not included in the study. Hence, potential differences in this patient group were not possible to investigate or analyze due lack of specific data.

The Swedish trauma registry was founded and implemented less than a decade ago. There may be a risk of missing patients due to lack of registration. The registry data were scrutinized for the three largest trauma centers in the country and potentially missed registered patients with firearm-related injuries were added. Furthermore, patients that had been registered twice due to transportation to a trauma centre were excluded. To adjust for variation in data quality and completeness, standardized data techniques were used, and missing values imputed by combining numeric and ordinal values. Nevertheless, some of the data analyzed were based on coding and might be subject to variations in practice. Registry data were combined with open access data from other authorities. The use of different data sources in parallel can provide a more complete understanding of the firearm injury epidemiology.

In conclusion, this study explored nationwide epidemiology of firearm injuries and assessed anatomical distribution of injuries, operative procedures, and outcomes. An overall increase of firearm injuries has occurred over the years, but may have plateaued in recent years. Firearm injuries remain an important public health problem and continue to cause significant morbidity and mortality. The lower extremity was the most common injury location but injuries to the head most lethal. The majority of injuries occurred in populated regions, but an increase was also seen in less urban areas which emphasize the importance of robust trauma systems with an appropriate field triage to transport patients to the nearest possible appropriate hospital. Given the economic and social impact of firearm injuries, as well as implementation of policy and public health efforts to reduce gun violence, additional population-based research is imperative.
